# David G. Watson (1934–2023)

**DOI:** 10.1107/S2056989023004723

**Published:** 2023-05-31

**Authors:** William Clegg

**Affiliations:** a School of Natural and Environmental Sciences, Newcastle University, Newcastle upon Tyne NE1 7RU, United Kingdom

**Keywords:** obituary

## Abstract

Obituary for David Watson.

I first met Dr David Watson when I was a postgraduate student doing research for my PhD under the supervision of Dr Peter Wheatley in Cambridge in the early 1970s. I was in a small laboratory in the short mezzanine corridor sandwiched between ground and first floors behind two large lecture theatres in the Lensfield Road Chemistry Departments, and the still rather young Cambridge Crystallographic Data Centre occupied offices in the same corridor, developing the Cambridge Structural Database. At the time I remember the staff included the Director Olga Kennard (herself recently deceased), Frank Allen and Sam Motherwell, all of whom I got to know. I remember David as a quiet, unobtrusive but dedicated and efficient scientist and I think he was effectively Olga’s second-in-command. David joined the CCDC when it was first founded in 1965 and contributed to its growth for over 30 years, during which he became head of the database team and moved with them to their new purpose-built home in 1992.

[Chem scheme1] 
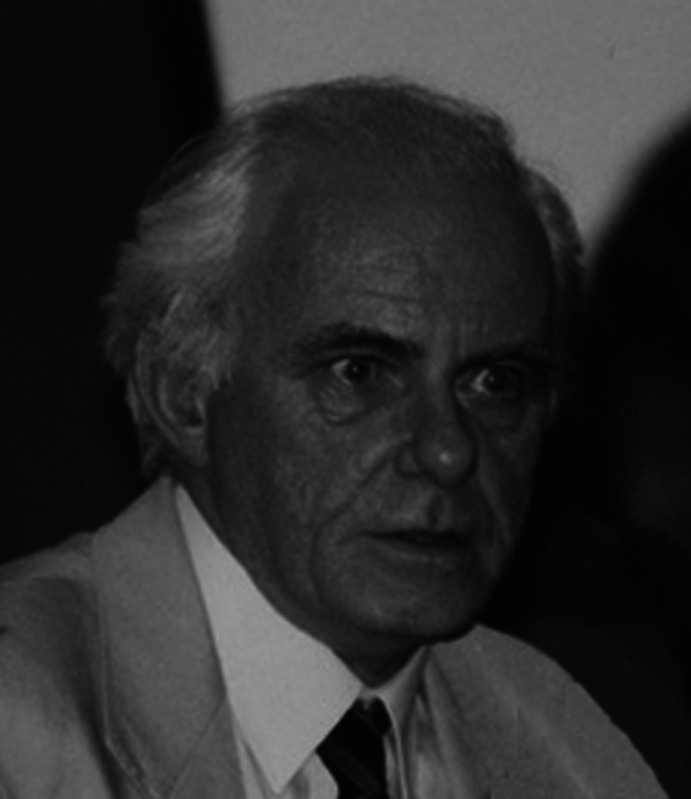


The CCDC has published an obituary focusing on David’s work there (https://www.ccdc.cam.ac.uk/discover/blog/obituary-dr-david-g-watson/), with tributes from some of his former colleagues. Gill Heale, CCDC Scientific Editor 1988–2007, adds: ‘David undertook my initial crystallographic training and it was a privilege to have such an excellent, methodical and patient teacher. We worked together on the data deposition scheme and it was a landmark day when we issued the first CCDC number. As our database group manager, David was kind, fair and approachable and I liked him very much.’ These are qualities he brought to the publishing activities of the IUCr.

Our paths next crossed in 2000, when the IUCr was planning the launch of its first non-print journal, *Section E* of *Acta Crystallographica*. The venture was strongly supported by the CCDC, represented mainly by Frank Allen. As this project would involve many new features and approaches, it was felt that *Section E* would benefit from having two Section Editors rather than the usual one. With a few years of experience as a Co-editor of *Section C* and a strong authorship record, I was invited to take up one role, and David was recommended by Frank for the other. We met for discussion in the editorial offices in Chester and quickly found we could work well together, having complementary backgrounds and common aims and standards. The journal was launched in 2001 and we served together until the 2008 IUCr Congress in Osaka, when the work was taken over by a team of three Section Editors. By then we had recruited a large and very international team of Co-editors.

David had an eye for detail, an excellent grasp of scientific writing style, and a desire for accuracy and consistency. These served him well in an editorial capacity. His proof-reading of articles was meticulous but also rapid, usually with a turnover of just hours for a large daily batch of manuscripts. He contributed enormously to the early success and productivity of *Section E* and to its establishment and development. When we both stepped down in 2008, I was glad of the released time for other activities, but David chose to continue in the team as a Co-Eeditor, which helped with continuity under the new leadership. He finally stepped down from the Editorial Board, after 12 years with the journal, in 2012.

He is remembered with great affection by the editorial staff in the IUCr’s Chester Offices. Peter Strickland, who was IUCr Managing Editor during the period that David was on the *Section E* board noted: ‘David was a meticulous planner and played a huge part in the setting up of *Section E* and making fellow crystallographers aware of the new journal. He was kind and friendly to all the staff at the IUCr, and we will miss him.’

David was one of the gentlest and most reliable scientists with whom I have ever had the privilege and pleasure of collaborating. I have fond memories of our shared experiences in this editorial work and I was saddened by the news of his death. His scientific legacy, both at the CCDC and for the IUCr, is exceptional and greatly valued.

